# The Blue Treasure: Comprehensive Biorefinery of Blue Crab (*Callinectes sapidus*)

**DOI:** 10.3390/foods13132018

**Published:** 2024-06-26

**Authors:** Elena Tamburini

**Affiliations:** Department of Environmental and Prevention Sciences, University of Ferrara, Via L. Borsari 46, 44121 Ferrara, Italy; tme@unife.it; Tel.: +39-0532-455172

**Keywords:** blue crab *Callinectes sapidus*, valorisation, by-products, shellfish, bioactive compounds

## Abstract

The blue crab, *Callinectes sapidus* (Rathbun, 1896), has become an invading species in the Mediterranean region, almost completely replacing native species within a few years and causing significant loss to local production. In some areas, there is an urgent need to propose new supply chains based on blue crab exploitation, where the potential valorisation routes for unsaleable blue crab and waste play an important role. The final purpose is to transform a threat into a treasure, towards a more sustainable world. In addition to applications in food industries, the considerable quantity of bioactive compounds in by-products, such as polysaccharides, proteins, amino acids, carotenoids, and chitin, needs to be capitalised by means of efficacious strategies and appropriate management. Crab exoskeleton can also be exploited as a carbonaceous material with applications in several fields, including medicine. Blue crab bioactive molecules have been widely recognised for having antioxidant, anticancer, antidiabetic, anti-inflammatory, and antimicrobial properties. Due to these functional and distinctive activities, such high-value components could be employed in various industries such as food–feed–pharma and cosmetics. Recycling and reusing these underutilised but economically valuable waste or by-products could help to reduce the environmental impacts of the whole supply chain from the perspective of the circular economy.

## 1. Introduction

The bio-refinery approach envisages bio-waste and by-products as potential renewable sources that can be valorised by means of the application of biotechnological processes to generate valuable bio-products and bioenergy comparable with fossil-based refineries, but with a significantly smaller environmental impact and hopefully reduced costs [1,2]. Bio-refineries, which target the valorisation of agriculture and aquaculture feedstock, have been recognised as a key driver towards a circular and sustainable economy [3]. They consist of several downstream strategies, i.e., extraction, refining, and purification, using biological methods for obtaining value-added biomolecules [4]. There has been an increasing interest in the utilisation of marine products, and novel bioprocessing technologies are developing for the isolation of some bioactive substances [5]. Fishery waste can become a viable and available source for biorefineries, whereby the biorefinery approach provides a low-cost conversion of fish waste into value-added products such as biofuels, industrial chemicals, animal feed, organic fertiliser, nutraceuticals, and others [6].

The blue Atlantic crab, *Callinectes sapidus* (Rathbun, 1986), is a decapod crustacean of the Portunidae family, largely diffused along the Atlantic and Gulf coasts, from Nova Scotia through the Gulf of Mexico and Uruguay, where it represents a commercially valuable shellfish product throughout [7]. *C. sapidus* is a euryhaline and eurythermal species capable of occupying several coastal habitats, such as estuaries and lagoons. It is characterised by high fecundity and aggressive behaviour, and its introduction into European and Asian seas, accidentally or intentionally, has taken place via commercial shipping activities, aquaculture, fisheries, and the aquarium industry [8]. Specifically, the species was first registered in the Mediterranean Sea in Greece, in 1948 [9]. Since then, population outbreaks have been reported in 15 lagoonal systems along the Turkish [10] coastlines, in North Africa [11,12], in the Black Sea [13], in the Ebro Delta [14], and throughout the Adriatic Sea as far as Grado and Marano lagoon, Gulf of Trieste [15], with recurrent findings in Croatia, Montenegro, Albania, and Italy [7]. In the last few years, Italian records on its occurrence were also reported at La Spezia harbour in the Ligurian Sea [16], on the eastern coast of Sicily [17], and along the whole Sardinian shoreline [18]. It is now evident that the blue crab has overgrown the trophic network through direct predation and through the bioturbation of the sediment [19]. 

Most of the early reports about the diffusion of *C. sapidus* on Mediterranean coasts refer to instances of incidental and limited capture [18]. In recent times, especially in the areas of recent colonization, its abundance has increased exponentially as to be considered invasive, triggering strong effects on autochthonous biodiversity (competition with native species, decline towards extinction of several species) and on local production, such as the Manila clam within the Sacca di Goro and Sacca degli Scardovari lagoons, northeastern Adriatic Sea [20]. 

The complete eradication of blue crabs currently represents an unrealistic scenario, and it is now well acknowledged that fishing for and using blue crab as a resource is the only way to regulate this invasion [21]. In fact, in its native regions, especially in the USA and Mexico, blue crab fisheries are still considered among the highest-value commercial activities [22,23]. Crab meat is a favoured food for its noticeable taste and flavour, as well as for its nutritional richness, being a source of high-quality proteins, unsaturated fatty acids, vitamins, minerals, and several bioactive components [24]. Due to these characteristics, crab by-products can also be used as food additives, as flavour strengtheners, and as raw materials for various crab-based products [25].

In addition, the crab processing industry generates a huge quantity of food-grade residues and by-products (i.e., shells, legs, guts, meat residues) [26], representing up to 85% of the total live weight of crab. Considering an average handpicked meat yield of 100 g/kg, blue crab fisheries worldwide produce a total of approximately 270,000 tons of post-processing waste [27]. This is especially problematic in countries in which a recovery/valorisation supply chain does not yet exist, where such waste is often landfilled or incinerated [28]. Therefore, alternatives for crab waste utilization and valorisation have been encouraged through environmental awareness and regulations [29].

Early literature on blue crab by-products and waste reutilization has shown their use as feeds in the poultry and cattle industries [30,31]. However, such low-cost applications have hindered other valuable potentialities that emerged later [32], and several studies have demonstrated that these by-products and waste utilization hide a treasure [33,34,35]. 

Our purpose was to resume the applications focusing on the valorisation of *C. sapidus* waste and by-products. Moreover, promoting the recovery of valuable compounds in these by-products can also bring additional economic and environmental benefits, as well as help achieve the Agenda 2030 goals [36]. Bioactive compounds from fishery sources have been already recognised and accepted by consumers in several sectors as natural sources, from food to pharma and cosmeceutical [37]. Of the few thousand scientific sources published in the 2000–2024 interval and collected from Google Scholar™, we have opted for 163 as references in the present review, including research articles, books, and reviews, only written in the English language, from internationally ranked sources and selected by the criteria of number of reported citations, scientific quality, and relevance of results. The search keywords were blue crab *Callinectes sapidus* waste valorization, invasive blue crab trends in valorization, bioactive compounds from blue crab *Callinectes sapidus* waste, and other keywords for the specific compounds (i.e., chitin, chitosan, astaxanthin) without inserting values between any Boolean operator, The method chosen to organise the information gathered from the literature is the semi-systematic review approach, as it is a useful tool to provide an overview of the research topics and to delineate the state-of-art, as well as its theoretical, perspectives [38].

The principal uses of blue crab waste can be resumed as animal feed, bioactive compounds extraction, carbonaceous material, and biofiller, comprehensively described in the following sections. Some small-scale and niche applications recently proposed will also be reported.

This review provides updated information on the possible valorisation of blue crab by-products, which will be especially relevant for the Mediterranean areas that have recently been invaded and that have an urgent need to handle this emergency and subsequently create new supply chains that offset the severe economic and productive damage suffered by their native species due to this invasion.

## 2. *C. sapidus* Product and Processing Overview 

In the global market, crab is available in frozen, fresh, and processed forms. According to EUMOFA [39], typical processing products include the following:

- Primary products, e.g., cooked whole or dressed crab;
- Secondary products, e.g., white picking meat (fresh, frozen, pasteurised, or canned);
- Tertiary products, e.g., white, brown, or mixed picking meat used as an ingredient in another product, including pâtés, pastes, and crab cakes.

White crab meat comes from most areas on its main body, including the breast and claws. Its texture is more meat-like and is a real hit as a top-shelf ingredient in seafood restaurants around the world, whereas brown crab meat is the liver or hepatopancreas [40]. 

**Figure 1:**
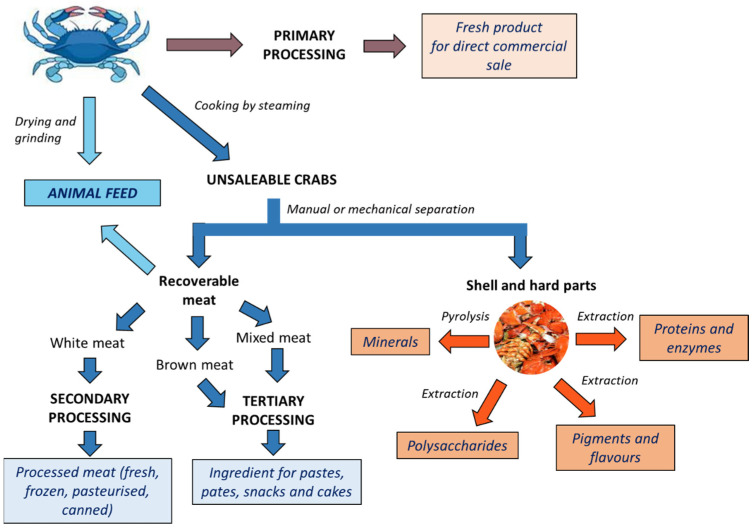
Different products from crab processing and valuable by-products from unsaleable crabs, including some of their applications in different fields.

Excluding primary products, which refers only to fresh, chilled, and frozen whole crabs, in picking plants, secondary and tertiary processing produce significant quantities of potentially recoverable food-grade by-products, such as shells, viscera, and residual meat remaining in the slabs and legs. Crab shells (exoskeleton) and hard parts (i.e., legs, claws) are also rich in precious bioactive molecules, such as proteins, minerals (mainly carbonates of calcium and magnesium), pigments, flavourings, and chitin (Figure 1).

Fresh live crabs are generally transported via boat or truck to manufacturing plants. A washing step is operated to remove fouling, sand, and grit. Then, crabs are cooked by steaming for a few minutes at 121 °C to facilitate meat removal from the shell (meat picking). Steam-processed blue crabs yield roughly 10% meat by weight when handpicked. Residues and waste are usually thrown away or further processed into low-value crab meal (tertiary processing) [41]. Mechanical extraction of minced meat from the by-products of handpicking operations could generate 15% to 20% more edible meat than that recovered via primary handpicking. Mechanised meat picking is usually carried out by means of hammer mills with brine-tank flotation, or high-speed vibration with a mechanical cutter which removes the legs, and spray jets and brushes which remove the carapace and viscera. After the picking process, the meat goes through a deboning step where any remaining shell fragments are eliminated. Then, the deboned meat is packed fresh, frozen, canned, or pasteurised to inactivate most of the spoilage microorganisms. 

The crab meat-based products derived from tertiary processing are principally produced using crab meat as a whole after separating residual meat from the exoskeleton, claws, legs, and body. A recent technique in tertiary processing utilises mechanically separated meat (MSM) machines, whereby the body of the crab passes between two rollers and the product is a paste comprising white and dark crab meat, where the meats of the thoracic sternum and the pereiopods are laminated together. Then, the paste is sent to a processing line for the withdrawal of the carapace, extraction of the viscera, and, finally, passage through the pulp that performs the pressing of the crustacean, resulting in a paste. The final product has a low commercial value owing to its slippery consistency, which renders it suitable only as an ingredient in fish sauces, pastes, pates, soups or cakes.

## 3. Composition of Crab Meat and Shell

The biochemical characterization of different crab tissues has been widely reported [42,43,44,45,46], and the proximate composition of the body and claw meat, as well as brown meat, of blue crabs from the Mediterranean areas is reported in Table 1 and Table 2.

The wide range of values shown is very likely due to the difference in areas of fishing and seasons, but the common trait is a high content of proteins and lipids. Furthermore, Table 1 shows that the lipid fraction is rich in polyunsaturated fatty acids (PUFAs), the human health benefits of which are well-known. In particular, as reported by Khamassi et al. [50], the long-chain omega-3 PUFAs eicosapentaenoic acid (EPA), docosahexaenoic acid (DHA), and arachidonic acid were the most abundant in all crab tissues, ranging from 9.3 to 15.6%, 8.7 to 13.7%, and 6.8 to 9.2%, respectively. EPA and DHA are referred to as ‘conditionally essential’ and are of special importance in the nutritional needs of young children and adolescents, as well as during pregnancy and at advanced age [51]. Crab meat is also rich in micronutrients, i.e., vitamins, especially folate, B-group vitamins, retinol (Vitamin A), ascorbic acid (Vitamin C), and minerals [52]. Vitamins are organic compounds that the body cannot synthesise on its own, participating in cell structure, energy metabolism, and all vital activities of the body. In particular, folate (Vitamin B9), as well as the other B-group vitamins, has a role in the prevention of neural tube defects in the foetus, in the proper functioning of the nervous and immune system, and in reducing the risk of developing certain cancers [53]. Retinol plays a crucial role in many physiological processes, including the growth and differentiation of target tissues, reproduction, proper functioning of the retina, and modulation of the immune system, and ascorbic acid is essential for collagen, carnitine, and neurotransmitter biosynthesis [54]. In addition, mineral nutrients are considered indispensable among human nutritional requirements, even though their daily uptake is small, particularly when compared with nutrients such as carbohydrates and lipids. They fulfil a wide variety of functions, such as building materials for our bones, influencing muscle and nerve function, and regulating the body’s water balance [55]. 

The protein content varies significantly depending on catch conditions (season and location), feeding, crab species, age, sex, etc. [56]. For example, the total essential amino acid (EAA) composition of blue crab meat ranged from 5 to 35 mg\100 g and included seven AAs (threonine, valine, methionine, leucine, phenylalanine, histidine, and lysine), whereas the total non-essential amino acid (NEAA) content was 20–40 mg\100 g [57].

The exoskeleton, as in all crustaceans, provides protection, support, and a site for muscle attachment for movement, and it also prevents water loss. Blue crab carapace is averagely composed of 70% organic matrix and 30% mineral that is mostly CaCO_3_ (75–80%), with smaller amounts of Ca_3_(PO_4_)_2_ (10–20%), MgCO_3_ (5–15%), and other crystals. The organic matrix consists of 30–40% protein and 20–30% chitin on a wet weight basis [58]. 

## 4. Use as Animal Feed

Historically, solid waste from blue crab processing plants has been sent to dehydration plants on a daily basis, where the waste was then dried and ground and sold as animal feed [59]. Crab meal generally contains approximately 35% crude protein and a high level of calcium, which makes it ideal as a calcium supplement for laying hens [60]. Cathcart et al. [31] suggested blue crab waste composting with chopped straw as a valuable disposal method, with the straw enabling the correct C:N ratio and moisture content to be obtained (estimated by the authors as 20:1 and 40–70%, respectively). In 35 days, the blue crab waste/chopped straw compost was stable and gave off few, if any, odours. There have been limited studies conducted evaluating the inclusion of crab and lobster meal into animal feed. The main concern with feeding crab and lobster meal to livestock is encouraging the animals to consume it, due to the limited palatability being derived from a large amount of chitins [61]. The flavour and overall desirability of cooked meat were not adversely affected by feeding ruminants up to 30% crab waste–straw silage [62].

Including low-cost alternative protein sources in fish feed has been a matter of feed research for an extended period. Crab wastes have potential as a replacement for fish meal if the shell and other hard parts are removed because chitin is indigestible to some fish and the high ash content can negatively impact feed absorption [63]. However, some studies on whole-crab meal digestibility in fish have reported a digestibility of 82% in haddock [64], 89% in cod [65], and 88% in Atlantic halibut [66]. The potential adverse effects of chitin and ash on digestibility by a species seem to be a function of specific stomach apparatus and digestion enzyme activities [67]. In fact, it has been found that fish that naturally feed on crustaceans show high levels of chitinase, suggesting the importance of chitinolytic enzymes in the fish digestion process [68]. Dean et al. reported a high feed conversion efficiency and weight gain in catfish fed with commercial fish meal added with 10% of crab waste without carapace. Autolyzed prawn and crab wastes have been utilised experimentally as a feed for seabass, but the results indicated that shellfish waste alone did not perform well regarding seabass nutritional quality, whereas a 1:1 (*w*/*w*) mixture of dried offal and shellfish waste performed better [69]. It was shown that crab meal served as a suitable partial replacement for tuna by-product meal and could improve the growth, feed conversion, and protein efficiency of cultured shrimp juveniles [70].

## 5. Extraction of Bioactive Compounds from Crab Shell Waste

Bioactive compounds potentially valorisable from blue crab waste are summarised in Table 3.

### 5.1. Polysaccharides

Chitin is one of the most abundant polysaccharide polymers after cellulose. Its content in *C. sapidus* shell may reach up to 70% on a dry weight basis, depending on the species and other factors [71]. Due to its non-toxic, antioxidant, biocompatibility, biodegradability, and renewability properties, chitin is used in many fields such as food, agriculture, cosmetics, and biotechnology [72]. In addition, due to their wide availability, low cost, and high biocompatibility, chitin and chitin-based polysaccharides from crab shell wastes are typically used as the basis for subsequent chemical or biotechnological modifications, as well as for copolymerization to improve material functionalities [24]. 

Several techniques are now available for chitin extraction from crab shell. The traditional chemical extraction process of chitin from dried and pulverised shells involves demineralization with dilute hydrochloric acid followed by deproteinization with dilute alkali and, finally, a decolouration, washing, and drying step [73]. The yield of chitin is about 25% of dry crab shell [74]. In addition, chitin can be deacetylated to chitosan using 30 to 60% (*w*/*v*) sodium or potassium hydroxide at 80–140 °C. The chemical methods and the high treatment temperatures have an influence on the molecular weight, degree of deacetylation, and functional properties of chitosan [75]. Chitosan has a huge number of applications compared to chitin because of the former’s higher solubility and biocompatibility [76]. In fact, it has been shown that chitosan and its further derivatives have several biomedical applications, including free radical scavenging and antihypertensive, anticoagulant, antidiabetic, anti-obesity, antiallergic, anti-inflammatory, antimicrobial, anticancer, and anti-Alzheimer effects [77]. The antibacterial and antifungal properties of chitosan qualify it for use in food packaging films and edible food coatings [78]. Its mechanical, gas, and water vapour permeability properties can be enhanced by blending chitosan with other natural polymers such as starch [79], cellulose [80], clay [81], silk fibroin [82], collagen [83], and pectin from orange peel [84], as well as with essential plant oils [85] or quinoa proteins [86]. Chitosan extracted from blue crab shell waste also represents a valuable substitute for synthetic antioxidants due to its high scavenging activity and an effective antimicrobial agent against common pathogenic microorganisms like *Escherichia coli* and *Staphylococcus aureus* [58]. Porous and nanofiber α-chitosan derived from blue crab have been effectively employed in biosensors, filtration (membrane), drug delivery, dental applications, and textile areas [87].

As an alternative to chemical processes, biological methods, such as enzymatic hydrolysis, have recently been proposed to extract chitin from crab shells. These processes provide better quality, require less energy, and consume less fresh water, unlike traditional ones [88]. Hajji et al. fermented crab shell waste using six protease-producing *Bacillus* bacteria to extract chitin [89]. Chitin extraction and lactic acid production from crab shells via the fermentation of *Lactobacillus* spp. using sugar cane molasses as a carbon source have been recently reported by Doan et al. [90]. Lactic acid fermentation has been reported by several authors as a beneficial alternative to chemical processes for extracting chitin from seafood [91,92]. Fermentation via lactic acid bacteria (LAB) has advantages over conventional methods for chitin extraction. One beneficial LAB is *Lactobacillus plantarum*, and other LAB include *L. paracasei*, *L. acidophilus*, *L. lactis*, *L. delbruekii*, *Serratia marcescens*, and *Bifidobacterium casei,* but the non-LAB *Aspergillus niger* is also used [93]. Fermentation for a maximum period of 7 days resulted in extensive deproteination and demineralization of crab shells, facilitating chitin recovery. The process can be conducted under conditions such as anaerobic, solid-stat, semi-continuous, or co-fermentation [36]. When enzymatic deproteinization is used, a previous demineralization can increase the enzyme permeability of the tissues and reduce the presence of potential enzyme inhibitors. Instead of the conventional hydrochloric acid, the use of organic acids (lactic and acetic) has been suggested; organic acids were shown to be comparable to hydrochloric acid but less harmful when helping to maintain the integrity of chitin [94]. In another study, it was shown that crude proteases from fish viscera can be used, thus lowering the cost of treatment [95]; the treatment caused up to 90% deproteinization, thereby facilitating the release of chitin.

Chitin and chitosan proved to be a versatile and promising biopolymer [96]. The use of these biopolymers is in various fields. They have an important role as natural alternatives having some biological properties and some specific applications like drug delivery, tissue engineering, functional food, food preservative, biocatalyst immobilization, wastewater treatment, molecular imprinting and metal nanocomposites [97]. The molecular mechanism of the biological properties, such as biocompatibility, non-toxicity, mucoadhesion, permeation enhancing effect, anticholesterolemic, and antimicrobial, has been an area of interest for many researchers [98]. In this regard, chitin, together with its derivative chitosan, is the most promising for this purpose due to its good film-forming abilities leading to the production of mechanically stable films and consequent utilization in the preparation of active film materials in food packaging [99].

Chitin collected from crab shell waste can also be processed into biopolymeric film together with coconut shell powder and castor oil [100], obtaining a product with improved mechanical and physicochemical properties like tensile strength, impact strength, elongation at break, water solubility, swelling, film thickness, and biodegradability.

### 5.2. Carotenoids, Flavours, and Pigments 

Aquatic animals, crustaceans in particular, are considered one of the most important sources of natural carotenoids. Astaxanthin, an oxygenated derivative of carotenoids and the main compound responsible for the orange-pink colour in the muscle of some crustaceans and salmonids, was found to be the major carotenoid in crabs. Astaxanthin has deserved appreciable attention due to its exceptional health properties such as its excellent antioxidant and anti-inflammatory activity. Consequently, it has become a highly demanded metabolite for its wide applications in the nutraceutical, cosmetic, food, and animal feed industries. Crabs, like other aquatic animals that contain considerable levels of astaxanthin, cannot synthesise astaxanthin but accumulate it via the ingestion of zooplanktons, which, in turn, feed on marine β-carotene-rich algae and convert β-carotene to astaxanthin in their bodies. Crustaceans accumulate astaxanthin in the exoskeleton, principally as crustacyanin, a carotenoprotein responsible for the red, purple, and blue to blue-black colour.

Conventionally, astaxanthin is extracted from exoskeleton wastes via solvent extraction, using food-grade acetone, benzyl alcohol, ethyl acetate, hexane, isopropanol, methanol, methyl ethyl ketone, and ethanol [101,102]. A more cost-effective and environmentally friendly approach was attempted using vegetable oil extraction [103], while Hooshmand et al. [104] obtained a high yield of extraction using sunflower oil, soybean oil, and rice bran oil, albeit always lower than that obtained with acetone extraction. Routray et al. studied the effect of acid ensilage treatment to increase the concentration of astaxanthin in oil recovered after extraction [101]. Cod liver oil has also been used to recover pigments from processing waste of snow crab and shrimp waste [105,106]. Studies have also been carried out on the recovery of carotenoids from crab carapace via supercritical CO_2_ extraction with co-solvents [107,108] or preceded by a microwave treatment on shell waste [109]. 

Alternative approaches have been addressed to microbial-assisted methods to facilitate astaxanthin recovery from exoskeleton waste using chitinolytic and proteolytic microorganisms, such as *Bacillus amyloliquefaciens*, showing remarkable protease and chitinase activity [110]. Hamdi et al. [111] suggested that pre-treatment using *Lactobacillus acidophilus* and *Saccharomyces cerevisiae* followed by solvent extraction with hexane and acetone at a ratio of 1:1 treatment led to the highest astaxanthin content. 

In addition to astaxanthin, investigations into the carotenoids of blue crab carapace have confirmed the presence of canthaxanthin, 4-hydroxiequinone, and 3-ketocanthaxanthin, which can be used as natural pigments in food and fish feed [112]. 

Crab exoskeleton is high in protein and can be treated with various proteases, thus obtaining free amino acids such as arginine, alanine, glycine, taurine, glutamic acid taurine, and glycine, which are primary taste-active components and major contributors to crab flavour [113]. The quantity and quality of free amino acids have a substantial function in augmenting the umami taste and improving food taste. Umami is the fifth basic taste of food together with salt, sweet, sour, and bitter and gives the main characterization to crab meat products [114]. Enzymatic proteolysis has been successfully applied by Moral et al. using bromelain, papain, and natural protease [112]. A crab extract or flavour could ameliorate the taste and flavour of an inexpensive seafood or could be used in further processed products such as crab cakes, stuffed crabs, crab stuffing and mixes, soups, bisques, and condiments for potato chips and dips. 

To date, shellfish protein hydrolysates have principally been employed for the production of low-value livestock and fish feeds, flavours, and additives for food. However, several studies have evidenced that marine-derived protein hydrolysates and peptides, including crab waste, can be utilised as functional food ingredients in the prevention of cardiovascular disease (CVD), diabetes, cancer, and obesity-related chronic conditions [115,116,117], owing to their antioxidant, antihypertensive, anticoagulant calcium-binding, appetite-suppressant, and human immunodeficiency virus (HIV)-1 protease-inhibitory activities [118]. Like that in other crustaceans, the haemolymph in blue crab contains biologically active substances such as lectins, clotting factors, and antimicrobial peptides, which have been shown to have a primary role in the innate immune defence system of marine invertebrates [119]. One such peptide, callinectin, represents the major antibiotic activity in blue crab and has been isolated from *C. sapidus* hemocytes [120]. Moreover, both peptides and amino acids extracted from blue crabs can act as biostimulants for plant growth and the synthesis of chlorophyll and for reducing the effects of abiotic stresses in vegetables [121].

## 6. Valorisation of Shell as Carbonaceous Material

Potential applications of blue crab exoskeleton waste as carbonaceous material are summarised in Table 4.

### 6.1. Applications as Bio-Absorbent 

Being rich in calcium carbonate, crab shells can be converted into biogenic adsorbent materials, templates for nanostructures, carbon sources of higher added value, or precursors for a calcium-rich biochar [122]. The principal methods for transforming crab shells into adsorbents are biochar production via pyrolysis, simply drying at low temperature (<100 °C), and grinding shells as they are [123]. Yao et al. reported that canal diameters of 40–50 nm make *C. sapidus* suitable for use as a sustainable and low-cost nanotemplate for nanostructured battery materials [124]. By means of scanning electronic microscopy, Cheng et al. [125] investigated the microstructure of *C. sapidus* exoskeleton to elucidate the mechanical behaviour of such biological composites [126].

One common carbon production technique is the fast pyrolysis process, which produces biochar from wastes and is considered an economical and environmentally friendly processing method for metal removal from wastewater [127]. The development of innovative high-added-value products such as pyrolyzed or activated carbons is possible due to the unique microstructure of crab shell [128]. Pyrolyzed carbons can be applied as filters to purify water or to eliminate the removal of dyes from effluents to the environment through the adsorption process [129]. Pyrolysis at 800 °C under a continuous flow of nitrogen of separated crab shells, pincers, and legs led to an average weight loss of about 50% and produced high-absorbance carbonaceous materials, tested on an artificial coloured solution of methylene blue, cationic malachite green, and anionic Congo red. The results showed that the carbonaceous material derived from crab legs has a maximum and quicker dye adsorption equal to 99.64% compared to pyrolyzed shell (90.30%) and pyrolyzed pincers (89.99%) [130].

Pyrolyzed *C. sapidus* exoskeleton powder showed a 90% efficacy towards the neonicotinoid pesticide acetamiprid absorption after a 15 min contact time [131]. 

Several applications have also been attempted using crab shell waste as a biomass adsorbent to remove heavy metals [96]. The bio-adsorption potential of *C. sapidus* dried shells for the removal of cadmium, nickel, and lead from hospital sewage was evaluated [132]. Therein, shells were simply dried and ground to a fine powder. A bio-adsorbent’s efficiency in heavy metal ion removal is a function of the porosity and functional groups (i.e., C-H, O-H, P-H, PO_4_^3−^ and C=O) on the adsorbent surface. Absorption capacities of crab shell powder in the range of 24–100% have also been achieved against six common antibiotics (i.e., tetracycline, norfloxacin, azithromycin, anhydroerythromycin, cephalexin, and amoxicillin), depending on the type and initial concentration.

The shells of blue crabs have been added to the anticancer drug 5-fluorouracil [133] and seaweed extract bio-fertiliser [134], featuring a mineral bio-composite powder produced from dried shells and organic active principles.

In a recent study, crab shells were utilised as sustainable solid adsorbents for CO_2_ capture, offering an environmentally friendly and low-cost alternative to conventional porous adsorbents such as zeolites, silicas, metal–organic frameworks, and porous carbons [135]. The natural porosity of crab shells has been fine-tuned by previous treatments of demineralization and deproteination to remove chitin and proteins [136]. The maximum CO_2_ uptake of crab-based materials has been found to be in the range of 0.13–0.22 mmCO_2_/g sample, with a progressive decline in absorption capacity during 10 cycles of absorption/desorption.

### 6.2. Applications in Medicine

The synthesis of biomaterials, such as hydroxyapatite or carbonated hydroxyapatite powders, as calcium carbonate-derived products derived from processed shells powders has been reported [137]. In addition, the materials obtained from crab shells are similar to human bones and have good biocompatibility with bones [138]. 

The benefits of employing biogenic instead of synthetic hydroxyapatite obtained from shell waste are lower costs of raw materials, no toxic chemicals used in production, more compatibility with native human hydroxyapatite, and higher cellular interaction, proliferation, and differentiation. Moreover, the biogenic hydroxyapatite production process, as compared to synthetic, guarantees a higher conversion rate and higher purity, with a low presence or absence of unconverted CaCO_3_ and/or other phases [139], which results in higher biocompatibility and bioactivity. In fact, in vitro studies have also confirmed positive results for shell sources with bone formation and regeneration when tested in vivo in mouse osteoclastic defect models [140]. 

Calcium from crab waste is also used as an element integrator in the pharmaceutical sector. In addition, shell wastes are used in medicine as reinforcing materials for the manufacture of prosthetic and implant materials [141]. In other work, Ri et al. [142] delivered calcium lactate and crab shell powder as a calcium supplement in the treatment of rickets in children. 

### 6.3. Applications as Bio-Catalyst

Calcium carbonate from crab shells can be calcinated to calcium oxide (CaO), a well-known catalyst for transesterification reaction for biodiesel production [143]. Calcination is the process of drying solids at high temperatures, typically 300–800 °C. During the calcination of the organic shells, carbon dioxide is removed from the carbonate structure such that the CaCO_3_ content is converted to the more catalytically active, porous CaO [144]. Jaekhajad and Nirusin [145] have recently investigated the synthesis of CaO from blue crab shells as a catalyst for biodiesel production from waste cooking oil by transesterification. Udomman et al. [146] have carried out a kinetic study of biodiesel production from palm oil by using low-cost CaO catalysts, prepared from eggshells, cockle shells, and crab shells, calcined at 800 °C. Xie et al. [147] evaluated the activity and the mechanisms of a crab shell bio-catalyst in the transesterification of rapeseed oil with methanol. Cardoso et al. [145] studied the influence of crab and mollusc shell residues with high calcium content as sources of high-activity heterogeneous catalysts for biodiesel production from soybean oil. Boey et al. [148] applied crab shell bio-catalyst to biodiesel production from chicken fat.

Additionally, a crab shell waste-derived carbon/CaO composite catalyst for biomass-derived glucose isomerization to fructose has been proposed [149,150].

## 7. Crab Shells as Bio-Filler for Polymers and Bio-Carbon Material

Dried and ground crab shells have recently found applications in the polymer industry as a bio-filler for polymers. Akbay et al. [151] proposed the incorporation of crab shell powder into ethylene propylene diene termonomer (EPDM) rubber. EPDM is present in many items of common use, such as cables, doors and windows, tubes, roofs, shoes, and high-tech applications [152,153]. Research has revealed that blue crab shells added to the EPDM matrix enhance the vulcanization parameters, tensile strength, and crosslinking density of composite material. Moreover, they facilitate a reduction in the amount of black carbon, one of the main components of EPDM, known for adverse health effects [154], including lung cancer [155]. Palaniyappan et al. [156] recently proposed the potential recycling and cleaner conversion of crab shell waste as a bio-reinforcement in the development of novel polylactic acid (PLA) biocomposite filaments for 3D-printing applications. The extruded filaments of PLA/crab shell (7%) have been used for the 3D-printing process via the fused filament fabrication method, showing better mechanical properties, extrudability, and printability compared with those of neat PLA. Polybenzoxazine resins, widely used as an anti-corrosive coating, adhesive, and flame retardants, have been reinforced with crab shell particles in the range of 0–30%, enhancing the polymerization, thermal stability, and mechanical characteristics [157].

Singaravelu et al. [158] recently developed brake pads for automotive applications, based on crab shell powder, resulting in better thermal stability, better heat dissipation, and coarse structure.

Crab shells can also be used as the raw material for the formulation of novel porous carbon materials for high-tech electronic applications. Shao et al. [159] produced a crab shell-derived nitrogen-doped micro-/mesoporous carbon as an effective separator coating for high-energy lithium–sulphur batteries [159]. Crab shell templates have been prepared to synthesise macroporous carbon nanofibers, obtaining materials with excellent capacitance properties and suitable for supercapacitor applications [160,161]. Fu et al. [162] used crab shells as porous carbon materials, as advanced electrode materials for supercapacitors, delivering excellent performance. Shi et al. [163] converted crab shells into 3D honeycomb-like graphitised hierarchically porous carbons, which presented a noteworthy level of capacitive performance as supercapacitor electrodes. The preparation of carbon materials from crab shells is clearly attracting more and more interest; however, studies so far have principally been carried out at the lab scale, requiring further studies for the higher-scale preparation of novel carbon materials.

## 8. Recovery of Bioactive Compounds from Crab Shell towards Zero-Waste Processing

**Figure 2:**
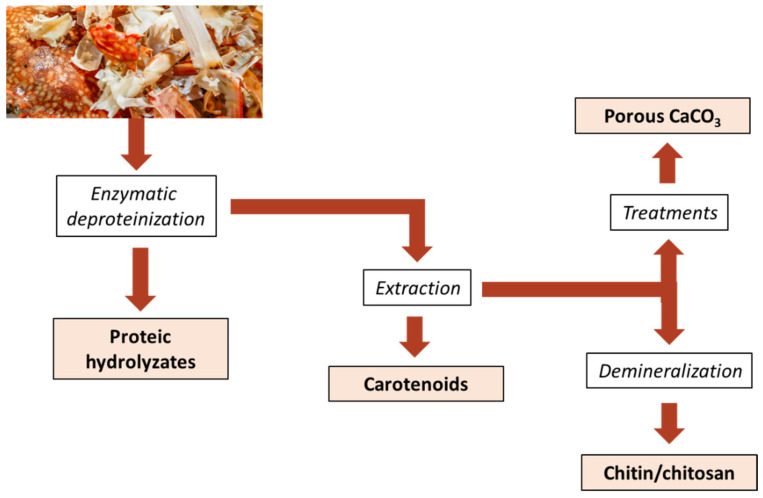
Scheme of the zero-waste valorisation for blue crab processing waste to obtain value-added products.

Antunes-Valcareggi et al. [164] proposed enzymatic hydrolysis with commercial proteases, i.e., alcalase and bromelain enzymes, as an eco-friendly approach to enzymatic deproteinization of crab wastes for producing high nutritional value hydrolysates (Figure 2). Enzymatic hydrolysis can be followed by solvent or supercritical CO_2_ extraction on the insoluble part to recover astaxanthin and other pigments in the form of a lipid–carotenoid extract.

Then, chitin was obtained from the insoluble fraction of astaxanthin recovery following demineralization in a 7% HCl solution and alkaline deproteinization. In general, chitin can also be extracted from calcium carbonate in the shell via a treatment with acid, e.g., citric acid, acetic acid, formic acid, and lactic acid, obtained from microbial fermentation.

Alternatively, the residual fraction from carotenoid extraction can undergo several treatments towards obtaining a biogenic nanoporous absorbent material for different applications, as reported in the previous section. According to Nekvapil et al. [131], a previous step of carotenoid extraction with solvents, followed by a mild thermal treatment and grinding of crab exoskeleton fragments, increased the powder porosity and, consequently, its absorbing activity.

These enzyme-based approaches are sustainable and eco-friendly in comparison to traditional methods and produce zero or near-zero waste, thus promoting the World Health Organization’s zero waste generation policy (recycling, and responsible production and consumption), while helping to generate additional revenue [165].

The scheme reported in Figure 1 highlights several biological routes for the recovery of valuable substances present in blue crab waste. Whereas single processes are unlikely to be cost-effective, an integrated biorefinery multiple-platform approach, through a cascade of various biotechnological processes, is more practical as part of a sustainable economy [36].

## 9. Conclusions and Implications of This Review

This review examines the potential valorisation routes for unsaleable blue crab and blue crab waste. Recently, this species has become invasive in Mediterranean areas; in some regions, it has almost completely replaced native species, not only crabs, with significant losses incurred by local fisheries and aquaculture production. The opportunity to consider crabs as a resource and to develop new supply chains based on primary processing and waste recycling is still challenging, and the necessary solutions should be adopted. Blue crab meat from secondary and tertiary processing can be reused as an ingredient for food preparation, and the exoskeleton contains several valuable bioactive compounds that can be extracted. In particular, the shell, legs, and claws are a source of polysaccharides such as chitin and chitosan, carotenoids, mainly astaxanthin, pigments, and flavours. They can also be used as a carbonaceous material with applications in several fields, including medicine.

A variety of innovative methods and processes are now being used to achieve a better bioactive compound extraction yield from crab shells. Furthermore, the building of biorefineries based on shellfish waste valorisation via microbial fermentation or through catalytic approaches to extract valuable chemicals and other materials is achieving more and more acceptance. The biorefinery approach to waste management can be cost-effective, eco-friendly, and sustainable compared to traditional methods, and it could be suitable for large-scale exploitation. Summing up, the ultimate aim of turning blue crab waste and by-products into value-added products can contribute towards reducing the pressure on the environment and solving landfill/disposal problems, while moving towards a more circular global economy and delivering a more sustainable future.

Of course, it is necessary to distinguish between regions where the blue crab is already a part of a well-established supply chain, like in the United States, and those where it represents an emerging outbreak, like the Mediterranean area. In the latter case, the primary processing chain and the entire waste/by-product valorisation supply chains have to be created de novo, including all the consequent financial and social difficulties. A preliminary techno-economic feasibility study is therefore essential in order to thoughtfully evaluate valorisation options, taking into account overall economic sustainability in terms of profit and investment. 

It may be more appropriate to implement a solution oriented towards the minimization of costs, with a minimal impact on the industrial plants and operations that have to be built, by initially using existing facilities or markets, while leaving niche and more extensive-investment applications, i.e., biomedical and pharmaceutical, to a later stage of development.

At present, special attention should be devoted to the invention and setup of advanced eco-friendly processes for the reuse of waste materials and preparation of active agents in sufficient quantities at relatively acceptable costs and low ecological footprint. Moreover, building a new supply chain is a multi-task problem that should be carefully considered and planned. 

It must also be said that the application of bioactive compounds from fishery sources is increasing daily to meet the demand of health-conscious consumers, and due to this increasing demand, this field is flourishing and requires further and advanced research. It is believed that the ocean will be the source of various medicinal products in the future, as seafood already provides thousands of valuable bioactive compounds for human use. To achieve a sustainable and hunger-free world, minimizing the discarding of food products is essential. From a food perspective, fishery by-products have tremendous potential for the blue economy and the initiative towards zero-waste management. More studies are therefore required to develop an innovative approach towards utilizing fishery discards and extracting their bioactive and nutritional compounds, which can ensure a circular economy not only in crab processing but in fishery-based industries as a whole.

In conclusion, this review article has dealt with a serious risk that hangs over the entire Mediterranean area, especially over some highly productive fishing and aquaculture areas, and has shown how it might be possible to turn this threat into an additional economic opportunity and resource that can be realised at the same time as part of blue economy and circular economy strategies. The suggestion that emerges is that, on the one hand, simpler and less costly forms of reuse should be used, such as use as animal feed, and, on the other hand, the possibilities of innovative processes in the field of extraction of bioactive compounds and the creation of new value chains with a zero-waste perspective should not be neglected. 

## Figures and Tables

**Table 1:** Proximate macro-nutrient composition of blue crab meats, expressed as a percentage (%) of fresh product.

Sample	Moisture (%)	Protein (%)	Lipids (%)	Ash (%)	Source
Body meat	66.4–86.7	14.7–30.3	0.4–1.6	1.1–2.0	[47,48,49]
Claw meat	65.6–83.1	15.0–31.0	0.6–1.2	1.3–2.1	[47,48,49]
Brown meat	73.6	18.8	0.9	2.15	[49]

**Table 2:** Proximate mineral composition of blue crab meats, expressed as mg/100 g of fresh product.

Sample	Na (mg/100 g)	K (mg/100 g)	Ca (mg/100 g)	Mg (mg/100 g)	P (mg/100 g)	Source
Body meat	326.9–723.6	62.8–244.4	64.9–455.4	37.1–85.5	165.4–202.2	[48,49]
Claw meat	266.8–663.9	69.1–256.3	149.2–398.2	35.1–117.7	135.2–176.2	[48,49]
Brown meat	1133.0	64.5	444.0	74.4	164.4	[49]

**Table 3:** Principal bioactive categories and molecules extractable from blue crab waste, including the main use and field of application. Corresponding references are reported within the main text for specific applications.

Bioactive Category	Bioactive Molecule	Use	Field of Application
Polysaccharides	Chitin	Bio-film	Food packaging Pharma-biomed
	Chitosan	Antioxidant Bio-film Bio-fibre	Food packaging Pharma-biomed
Carotenoids	Astaxanthin	Antioxidant Pigment	Food/feed additive
	Canthaxanthin, 4-hydroxiequinone, 3-ketocanthaxanthin	Natural pigments	Food/feed additive
Protein	Free amino acids, i.e., arginine, alanine, glycine, taurine, glutamic acid taurine, glycine	Flavour Plant biostimulant	Food/feed additive Agriculture
	Hydrolyzates	Flavour Functional ingredient	Food additive

**Table 4:** Principal applications of blue crab exoskeleton as carbonaceous materials, including the main uses and field of application. Corresponding references are reported within the main text for specific applications.

Application	Use	Field of Application
Bio-sorbent	Wastewater treatment	Metal removal Antibiotics removal
	CO_2_ capture	Industry
Biogenic hydroxyapatite	Implant material	Medicine
	Calcium supplementary	Pharmaceutical
Bio-catalyst	Biodiesel production	Biofuels
	Monosaccharides isomerization	Food
Bio-filler	Polymer additive	Industry
	Filaments	3D printing
	Brake pads	Automotive
	Porous carbon material and nanofibers	High-tech electronic

## Data Availability

Not applicable.
